# An analysis of muscle growth after proton beam therapy for pediatric cancer

**DOI:** 10.1093/jrr/rrad105

**Published:** 2024-01-23

**Authors:** Hazuki Nitta, Masashi Mizumoto, Yinuo Li, Yoshiko Oshiro, Hiroko Fukushima, Ryoko Suzuki, Sho Hosaka, Takashi Saito, Haruko Numajiri, Chie Kawano, Satoshi Kamizawa, Kazushi Maruo, Hideyuki Sakurai

**Affiliations:** Department of Radiation Oncology, University of Tsukuba, 1-1-1 Tennoudai, Tsukuba, Ibaraki 305-8575, Japan; Department of Radiation Oncology, University of Tsukuba, 1-1-1 Tennoudai, Tsukuba, Ibaraki 305-8575, Japan; Department of Radiation Oncology, University of Tsukuba, 1-1-1 Tennoudai, Tsukuba, Ibaraki 305-8575, Japan; Department of Radiation Oncology, Tsukuba Medical Center Hospital, 1-3-1 Amakubo, Tsukuba, Ibaraki, 305-8558, Japan; Department of Pediatrics, University of Tsukuba Hospital, 2-1-1 Amakubo, Tsukuba, Ibaraki, 305-8576, Japan; Department of Child Health, Institute of Medicine, University of Tsukuba, 1-1-1 Tennoudai, Tsukuba, Ibaraki 305-8575, Japan; Department of Pediatrics, University of Tsukuba Hospital, 2-1-1 Amakubo, Tsukuba, Ibaraki, 305-8576, Japan; Department of Child Health, Institute of Medicine, University of Tsukuba, 1-1-1 Tennoudai, Tsukuba, Ibaraki 305-8575, Japan; Department of Pediatrics, University of Tsukuba Hospital, 2-1-1 Amakubo, Tsukuba, Ibaraki, 305-8576, Japan; Department of Radiation Oncology, University of Tsukuba, 1-1-1 Tennoudai, Tsukuba, Ibaraki 305-8575, Japan; Department of Radiation Oncology, University of Tsukuba, 1-1-1 Tennoudai, Tsukuba, Ibaraki 305-8575, Japan; Department of Radiation Oncology, University of Tsukuba, 1-1-1 Tennoudai, Tsukuba, Ibaraki 305-8575, Japan; Department of Radiation Oncology, University of Tsukuba, 1-1-1 Tennoudai, Tsukuba, Ibaraki 305-8575, Japan; Department of Biostatistics, Institute of Medicine, University of Tsukuba, 1-1-1 Tennoudai, Tsukuba, Ibaraki 305-8575, Japan; Department of Radiation Oncology, University of Tsukuba, 1-1-1 Tennoudai, Tsukuba, Ibaraki 305-8575, Japan

**Keywords:** proton beam therapy, pediatric tumor, muscle, late effects, radiotherapy

## Abstract

Retardation of growth and development is a well-known late effect after radiotherapy for pediatric patients. The goal of the study was to examine the effect of proton beam therapy (PBT) on the growth of muscles included in the irradiated area. The subjects were 17 pediatric patients (age ≤ 5 years) who received PBT with a treatment field including a muscle on only one side out of a pair of symmetrical bilateral muscles and had imaging evaluations for at least 1 year after PBT. The thicknesses of the irradiated and non-irradiated (contralateral) muscles were measured retrospectively on CT or MRI axial images collected before and after PBT. The change of thickness divided by the period (years) for each muscle was compared between the irradiated and contralateral sides. Correlations of muscle growth with irradiation dose and age at the start of treatment were also evaluated. The median observation period was 39.2 months. The measurement sites included the erector spinae (*n* = 9), gluteus maximus (*n* = 5) and rhomboids + trapezius (*n* = 3) muscles. The average changes in muscle thickness were 0.24 mm/year on the irradiated side and 1.19 mm/year on the contralateral side, showing significantly reduced growth on the irradiated side (*P* = 0.001). Younger patients had greater muscle growth. Irradiation dose was not significant, but muscle growth tended to decrease as the dose increased, and muscles irradiated at >50 Gy (RBE) showed little growth. These results show that muscle growth is affected by PBT and that long-term follow-up is needed to evaluate muscle growth retardation.

## INTRODUCTION

Multimodal treatment of childhood tumors with surgery, chemotherapy and radiotherapy has improved the 5-year overall survival rate to around 80% [1]. However, the risk of late adverse events has become more of a concern with improved outcomes [2]. The energy peak of a proton beam results in an excellent dose distribution with a small number of ports, and the lower dose area can be reduced compared to photon radiotherapy. Therefore, proton beam therapy (PBT) is commonly used for pediatric tumors based on an expected reduction in late toxicities and secondary cancers [3]. However, it is still difficult to avoid effects on normal tissue in the irradiated area with PBT, and we have found that growth of vertebral bone in this area is reduced in a dose-dependent manner after PBT [4–6]. Growth disturbances in the vertebral bodies result in short stature and scoliosis [4–6]. Similarly, photon radiotherapy is known to cause growth disturbances in the muscles [7, 8]. Muscle growth disturbances result in facial asymmetry [9–14], scoliosis and other cosmetic problems [6, 15–17]. After radiotherapy for head and neck cancer in children, facial asymmetry occurs in 70–80% [10–12]. Although growth retardation is expected in PBT as well as in photon radiotherapy within the irradiated field, there have been no reports yet on the quantitative evaluation of muscle growth retardation. In this study, we examined the effect of PBT on growth of muscles included in the irradiated area.

## PATIENTS AND METHODS

The subjects were 17 patients among 249 pediatric cases treated with PBT at our center from November 2013 to May 2019. The inclusion criteria were age ≤ 5 years, a treatment field on one side including one of a pair of symmetrically bilateral muscles, and imaging evaluation for at least 1 year after PBT. The characteristics of the 17 patients (9 boys, 8 girls) are shown in Table 1. The median age was 3 years (range: 2–5 years); the diseases were neuroblastoma (*n* = 8), Ewing sarcoma (*n* = 3), rhabdomyosarcoma (*n* = 2), Wilms’ tumors (*n* = 2), yolk sac tumor (*n* = 1) and pleuropulmonary blastoma (*n* = 1); and the median irradiation dose was 30.6 Gy (relative biological effectiveness, RBE) (range: 10.8–61.2).

**Table 1:** Patient characteristics

Characteristics	*n* = 17
Age (years)	3 (median)
	2–5 (range)
Gender	
Male	9
Female	8
Disease	
Neuroblastoma	8
Ewing sarcoma	3
Rhabdomyosarcoma	2
Wilms tumor	2
Yolk sac tumor	1
Pleuropulmonary blastoma	1
PBT dose (Gy (RBE))	30.6 (median)
	10.8–61.2 (range)

Thicknesses of the irradiated and non-irradiated (contralateral) muscles were measured on computed tomography (CT) or magnetic resonance imaging (MRI) axial images obtained before and after PBT. The cross section to be measured was determined for each patient using anatomical structures such as vertebrae and blood vessels as the merkmal. Images of the same modality and under the same conditions such as slice thickness and contrast were used for each patient to reduce measurement error. If the tumor was intramuscular in the treatment field, the tumor-free area was chosen as the measurement site. The change per year was calculated as the change in muscle thickness divided by the period (years) for each side. A Wilcoxon signed-rank test implemented in SPSS ver. 28 (IBM, Co., New York, NY) was used to evaluate the difference in the change per year between the irradiated and contralateral sides, with *P* < 0.05 considered to be significant. Correlations of the differences in the change per year between the irradiated and contralateral sides with the irradiation dose and age at the start of treatment were examined using Spearman rank correlation coefficient analysis, with *r* > 0.4 defined as a correlation. The average of the difference in the change per year between the irradiated and contralateral sides was compared between patients who had surgery and those who did not. A Mann–Whitney U-test was used to evaluate the difference, with *P* < 0.05 considered to be significant.

The study was conducted in accordance with the Declaration of Helsinki and approved by the Institutional Review Board (or Ethics Committee) of Tsukuba Clinical Research & Development Organization (H30–099, 14 November 2019).

## RESULTS

**Fig. 1:**
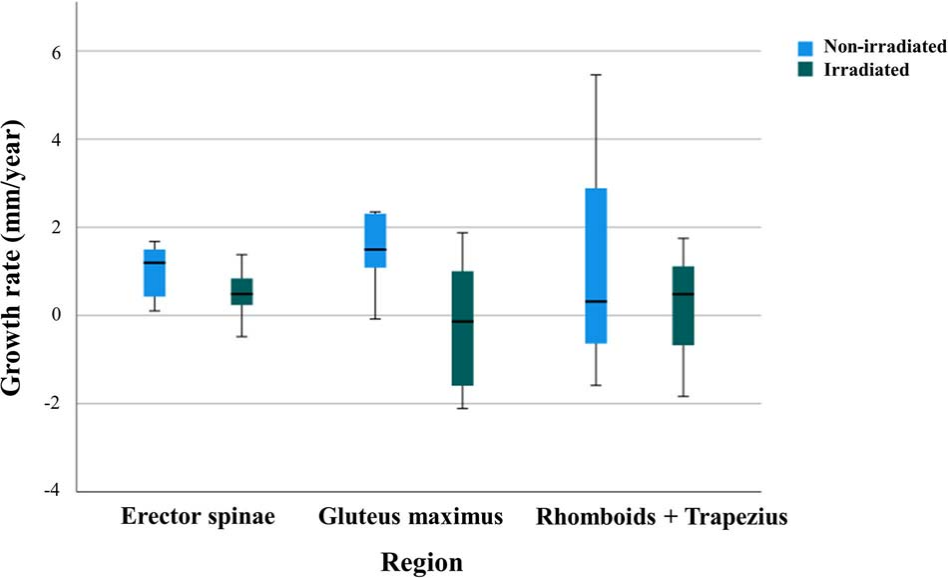
Changes in muscle thickness (mm/year) on the irradiated side (right) and non-irradiated (contralateral) side (left) at different muscle sites.

**Fig. 2:**
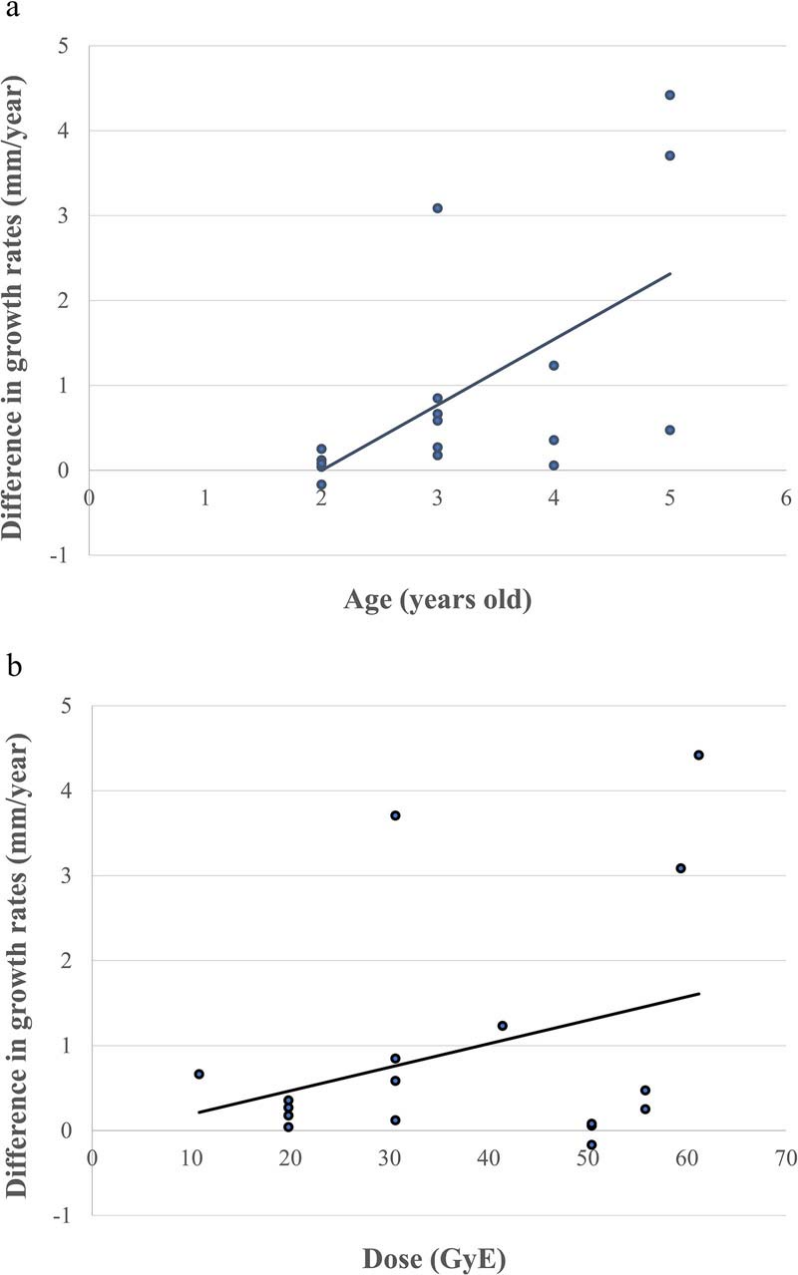
Relationships of changes of muscle thickness between the irradiated and contralateral sides with (**a**) age and (**b**) PBT dose.

**Fig. 3:**
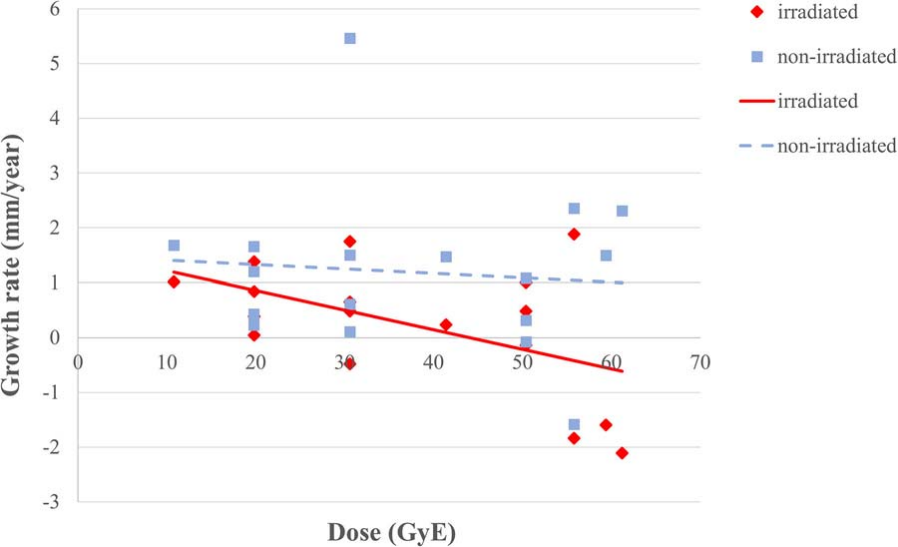
Changes of muscle thickness on the irradiated side (solid line, diamond shape) and non-irradiated (contralateral) side (dashed line, square shape) with PBT dose.

The median observation period was 39.2 months (range: 12.5–59.2 months). The measurement sites were the erector spinae (*n* = 9), gluteus maximus (*n* = 5) and rhomboids + trapezius (*n* = 3) muscles. Twelve patients underwent tumor resection prior to PBT and one patient underwent residual tumor resection after PBT. The average changes in muscle thickness were 0.24 mm/year (range: −2.11 to 1.88 mm/year) on the irradiated side and 1.19 mm/year (range: −1.58 to 5.46 mm/year) on the contralateral side, showing significantly worse growth on the irradiated side (*P* = 0.001). The mean changes for the erector spinae, gluteus maximus and rhomboids + trapezius muscles (irradiated vs contralateral) were 0.51 vs 0.99, −0.19 vs 1.43 and 0.13 vs 1.40 mm/year, respectively (Table 2, Fig. 1). For operated and non-operated cases, the mean changes (irradiated vs contralateral) were 0.49 vs 1.30, −0.38 vs 0.93 mm/year. According to a Mann–Whitney U-test, there was no difference between operated and non-operated cases (*P* = 0.195). Age was the only factor correlated with the difference in the change in muscle thickness, with younger patients showing better growth (Fig. 2). Irradiation dose was not significant, but muscle growth showed a tendency to decrease as the dose increased (Fig. 3). The growth rates at 20 Gy (RBE) and 50 Gy (RBE) (irradiated vs contralateral) were 0.86 vs 1.33 and − 0.21 vs 1.09 mm/year, respectively.

**Table 2:** Average and range of changes in muscle thickness by site

Site	Number of cases	Average (mm/year)		Range (mm/year)	
		Irradiated	Non-irradiated	Irradiated	Non-irradiated
Erector spinae muscle	9	0.51	0.99	−0.48 to 1.38	0.11 to 1.68
Gluteus maximus muscle	5	−0.19	1.43	−2.11 to 1.88	−0.08 to 2.35
Rhomboid major muscle + trapezius	3	0.13	1.40	−1.83 to 1.75	−1.58 to 5.46

**Fig. 4:**
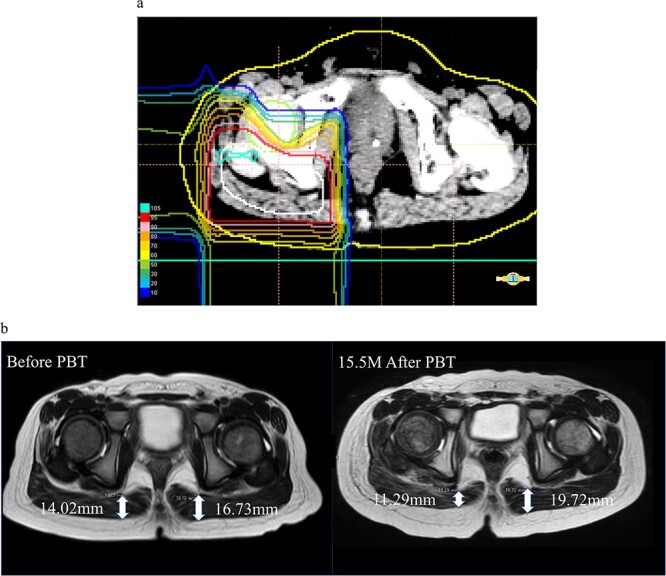
Case of a 5-year-old boy with Ewing’s sarcoma of the right hip. (**a**) Dose distribution of PBT. (**b**) Thickness of the irradiated and contralateral side muscles on MRI before PBT (left) and MRI at 15.5 months after PBT (right). The same cross-section is shown in the images before and after PBT.

An illustrative case is shown in Fig. 4. The patient was a 5-year-old boy with Ewing’s sarcoma of the right hip who received 61.2 Gy (RBE)/34 Fraction to the primary tumor (Fig. 4a). The thicknesses of the muscles before PBT were 14.02 mm on the irradiated side and 16.73 mm on the contralateral side (Fig. 4b, left). At 15.5 months after PBT (at age 6 years), these thicknesses were 11.29 and 19.72 mm, respectively, giving growth rates of −2.11 and 2.31 mm/year on the irradiated and contralateral sides, respectively (Fig. 4b, right).

Symptomatic late adverse event due to muscle growth disturbance was observed in only one patient. The patient was a 5-year-old girl who had received 55.8 Gy (RBE)/31 Fraction to the right pelvis (no surgery was performed) and had discomfort when walking due to shortening of the right lower limb length. No muscle late adverse events requiring treatment were observed.

## DISCUSSION

The prognosis of pediatric cancer has improved, but late effects have become a concern. Prediction and reduction of these effects are important issues. In pediatric patients, late effects after radiotherapy vary depending on the irradiation site. Growth and development retardation are well known late effects in these patients, and are affected by age and dose [18]. Growth retardation of the bone causes short stature [5], limb extension differences [19] and facial deformity [10–14]. Effects of radiotherapy on muscle growth may also occur [7, 8, 20, 21] due to muscle atrophy and fibrosis [8]. Guyuron *et al*. suggested that such effects could be caused by a dose of as little as 4 Gy [20]; however, generally, younger age and doses >20 Gy are considered to influence muscle growth [8, 21]. Reduced muscle growth on the face may cause facial deformity, and subsequent plastic surgery may be required. In a study of children aged 2–11 years with head and neck rhabdomyosarcoma, Paulino *et al*. reported development of facial asymmetry in 11 of 15 children (73%) in whom the irradiated field included part of the face, with 3 patients requiring reconstruction [12]. Also, growth disturbance of the paraspinal muscles may cause scoliosis as well as growth retardation of the vertebral bodies [6, 15–17].

PBT has good dose-localization compared to photon radiotherapy. Proton beams can reduce lower dose area and avoid adjacent risk organs with small number of ports. PBT is now widely used for pediatric patients as an alternative to photon radiotherapy because PBT reduces occurrence of secondary cancer by reducing the lower dose area, and PBT is expected to deliver high doses to tumors with fewer late toxicities especially for liver and esophageal cancer [22, 23]. However, adverse effects do not differ between photon radiotherapy and PBT in the treatment field where the same dose is delivered, and the steep fall-off in proton beams is likely to cause differences between irradiated and non-irradiated areas. Careful attention should be paid to avoid kyphosis and lordosis caused by proton beams stopping in the vertebral body [4, 24]. Growth effects in the treatment field also occur in PBT. In a study of vertebral growth disturbance caused by PBT, Baba *et al*. measured the height of 353 vertebral bodies irradiated with different doses in 23 patients, and found that the growth rate of the vertebral bodies decreased with increasing dose [4]. Also, Li *et al*. examined renal late effects after PBT for pediatric cancer and found a significantly reduced kidney volume on the irradiated side compared to that on the contralateral side [25].

The current study is the first quantitative evaluation of muscle growth disturbance associated with PBT. A comparison of the thickness of irradiated and non-irradiated bilateral muscles showed significantly reduced muscle growth on the irradiated side. The gluteal muscles showed the greatest growth reduction after PBT, which may be because these muscles have a larger volume and higher load area than those of the erector spinae and trapezius muscles. Some authors suggested that surgery also caused muscle growth retardation. In this study, however, muscle growth rate was similar among patients who received surgery and who did not receive surgery (*P* = 0.195). Age was a significant factor in growth difference, with muscle growth decreasing with increasing age. Younger age has previously been linked to severe muscle growth disturbance. Dorr *et al*. suggested that patients under 6 years old were at greatest risk for reduced muscle growth [7]; Lockney *et al*. found that patients with severe facial deformity had a median age at treatment of 6.0 years [14]; and Li *et al*. found that renal atrophy was more pronounced at age 4–7 years [25]. In this study, patients aged 2–5 was selected because these patients have steeper growth curve and we consider that the trends of muscle retardation would be more elucidated for younger children. Therefore, it is unclear if our findings are due to the small number of patients, or whether older patients within the 2–5 age range are more prone to muscle growth reduction. We found no correlation between reduced muscle growth and irradiation dose. However, muscle growth tended to be lower as the dose increased, and the mean growth rate in cases irradiated with ≥50 Gy (RBE) was −0.33 mm/year, which suggests that muscle growth is unlikely after irradiation at this dose, as well as bone growth [4, 6].

This study has number of limitations: small number of patients, the short follow-up period and uncertainty of measurement strategy of only length with one direction. However, this study suggested that muscle growth was significantly impaired by PBT. The effects of age and doses are future problem.

## CONFLICT OF INTEREST

The authors have no conflicts of interest to declare.

## FUNDING

None.

## ETHICS APPROVAL

The institutional review board approved this study (H30–099).
